# Changes in Membrane Protein Structural Biology

**DOI:** 10.3390/biology9110401

**Published:** 2020-11-16

**Authors:** James Birch, Harish Cheruvara, Nadisha Gamage, Peter J. Harrison, Ryan Lithgo, Andrew Quigley

**Affiliations:** 1 Membrane Protein Laboratory, Diamond Light Source Ltd., Harwell Science and Innovation Campus, Didcot OX11 0DE, UK; james.birch@diamond.ac.uk (J.B.); harish.cheruvara@diamond.ac.uk (H.C.); nadisha.gamage@diamond.ac.uk (N.G.); peter.harrison@diamond.ac.uk (P.J.H.); Ryan.Lithgo1@nottingham.ac.uk (R.L.); 2 Research Complex at Harwell (RCaH), Harwell Science and Innovation Campus, Didcot OX11 0FA, UK; 3 School of Biosciences, University of Nottingham, Sutton Bonington Campus, Loughborough LE12 5RD, Leicestershire, UK

**Keywords:** membrane protein, structural biology, crystallography, electron microscopy

## Abstract

**Simple Summary:**

Membrane proteins are essential to all forms of life. Millions of membrane proteins are found in the lipid membrane layer that surrounds cells, and in the lipid membrane layers that surround smaller cellular compartments. Many medicines interact with membrane proteins; these include drugs that treat cancer, heart disease and pain. Research into membrane proteins is therefore important to the design and development of new medicines. Membrane proteins are difficult to work with, partly because they are so small. However, using techniques such as X-ray crystallography and electron microscopy, structural biologists, including those at the Membrane Protein Laboratory, are able to see the atomic detail of membrane proteins. There has been great progress in the field of membrane protein structural biology over the past fifteen years. Here, we review the recent advances in membrane protein structural biology, highlight key methods and give an overview of techniques. We also discuss the challenges that remain in this field, and suggest areas for future research.

**Abstract:**

Membrane proteins are essential components of many biochemical processes and are important pharmaceutical targets. Membrane protein structural biology provides the molecular rationale for these biochemical process as well as being a highly useful tool for drug discovery. Unfortunately, membrane protein structural biology is a difficult area of study due to low protein yields and high levels of instability especially when membrane proteins are removed from their native environments. Despite this instability, membrane protein structural biology has made great leaps over the last fifteen years. Today, the landscape is almost unrecognisable. The numbers of available atomic resolution structures have increased 10-fold though advances in crystallography and more recently by cryo-electron microscopy. These advances in structural biology were achieved through the efforts of many researchers around the world as well as initiatives such as the Membrane Protein Laboratory (MPL) at Diamond Light Source. The MPL has helped, provided access to and contributed to advances in protein production, sample preparation and data collection. Together, these advances have enabled higher resolution structures, from less material, at a greater rate, from a more diverse range of membrane protein targets. Despite this success, significant challenges remain. Here, we review the progress made and highlight current and future challenges that will be overcome.

## 1. Introduction

Membrane proteins (MPs) are located in cellular and organellular membranes as well as the external layers of enveloped viruses. MPs have both structural and functional roles that include energy metabolism, signal transduction, transport, the immune response as well as many enzymatic processes. Over 50% of small-molecule drugs target human MPs despite MPs accounting for around 30% of the human proteome. The G-protein coupled receptor (GPCR) superfamily is the most targeted followed by ion channels; however, recent efforts to find new drug targets have led to a focus on solute carriers [1,2,3]. Our understanding of MP drug targets is facilitated by structural biology that informs on the conformational and mechanistic dynamics of these proteins as well as drug binding modes.

**Figure 1:**
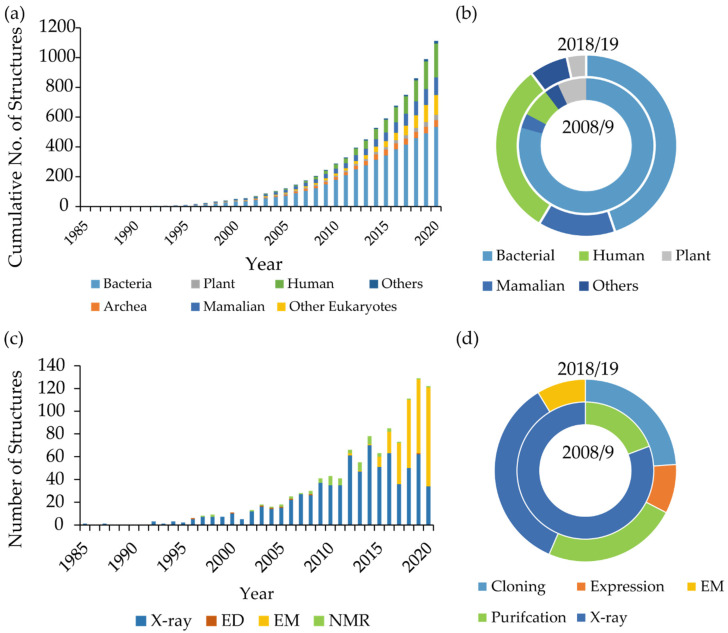
(**a**) Cumulative tally of unique membrane protein structures deposited in the Protein Data Bank (PDB) adapted from the Membrane Proteins of Known 3D Structure database [4]. (**b**) Membrane Protein Laboratory (MPL) projects classified by target origin in 2008/9 (29 projects) and 2018/19 (46 projects). (**c**) Number of membrane protein structures solved each year by electron diffraction (ED), electron microscopy (EM), X-ray crystallography and nuclear magnetic resonance spectroscopy (NMR) (**d**) accepted MPL projects by requested technology platform in 2008/9 (29 projects) and 2018/19 (46 projects). Cloning and expression were not supported in 2008/9.

The last fifteen years have witnessed a rapid expansion in MP structural biology. Multiple technological and methodological developments have led to 2588 coordinate files of MPs deposited in the Protein Data Bank (PDB). The “Membrane Proteins of Known 3D Structure” database currently (September 2020) lists 1141 unique MP structures (Figure 1) [4]. This contrasts with only 148 unique structures in 2008 close to when the Membrane Protein Laboratory (MPL) was established. Of these, 39 were of eukaryotic MPs and included just four human structures [5]. MPs continue to be among the more technically challenging proteins to work with. However, dedicated microfocus beamlines for macromolecular protein crystallography, associated advances in automated data processing pipelines as well as the resolution revolution in cryo-electron microscopy (cryo-EM) have shifted the challenge towards sample preparation [6,7,8,9].

The MPL was established in 2007 and has supported MP research projects, which have led to 29 depositions in the PDB (Table 1). Further work by scientists at the MPL has advanced MP production, crystallography and data processing [10,11,12,13,14,15,16]. During the last decade, there has been a shift towards solving structures of eukaryotic, mainly human, MPs. When the MPL was established, eukaryotic MPs accounted for approximately 30% of solved structures while human MPs accounted for just 5%. This trend is also reflected in a 450% increase in MPL applications associated with human MPs (Figure 1).

Initially, the MPL was focused on supporting projects with protein crystals or purified protein that was ready for crystallisation. However, it became apparent from rejected applications and multiple enquiries that there was a need to support researchers who were having difficulties with expressing and purifying MPs. More than 50% of our visiting scientists now require assistance with MP production (Figure 1). MP production is difficult due to low levels of expression, solubility and protein instability outside of the membrane. It is also difficult to obtain well-diffracting protein crystals or pure and stable samples for single-particle cryo-EM. Many tools have been developed to address these problems that include different optimised expression systems, MP solubilising agents and purification techniques, but it is not always clear which tools to use. Thus, an iterative screening approach is required that can be time and resource hungry. High throughput (HTP) screening strategies that quickly assess protein quality and stability go some way towards mitigating this.

This review highlights the major technological and methodological developments that have led to improvements in recombinant MP production, sample preparation and advances in data collection. It is not possible in the context of this review to give a detailed overview of every advance, and we encourage readers to supplement their reading with referenced review articles. We also discuss common issues and highlight methodologies that have been useful to MPL scientists.

## 2. Recombinant Membrane Protein Production

Recombinant production of MPs is the most favoured approach due to the scarcity of naturally available MPs. A strategy for successful MP production must consider a wide range of parameters that encompass construct design, expression system, extraction and purification tools. Generally, successful MP production strategies still rely on a trial and error approach. Bioinformatic servers and web-based databases are important resources when considering construct design, while a wide range of expression vectors with different reporter proteins and tags enable multiple purification options. Overexpression of an MP is often toxic, and matching the target MP origin with the most closely related expression system can improve production chances.

### 2.1. Generating Constructs for Recombinant Protein Expression

The first step is the design of protein constructs using prior knowledge of the protein target, good bioinformatics analyses of the sequence and giving thought to the intended experimental applications of each construct. The PSI-PRED workbench has many useful tools that encompass multiple analyses, summarised by a user-friendly web interface [32]. These tools include a prediction of secondary structure (PSIPRED), a protein disorder prediction (DISOPRED), a domain boundary prediction (DOMPRED), membrane helix prediction (MEMSAT-SVM) and protein fold recognition (GeneTHREADER) [33,34,35,36,37]. Construct boundaries that are likely to have a minimal effect on protein function can be predicted using information from the PSI-PRED workbench, BLASTp searches against the PDB and sequence annotations from UniProt, such as disulphide linkages, glycosylation and lipidation sites. Typically, 6–12 constructs per MP target will increase the likelihood of successful MP production and an atomic resolution structure [38,39].

Codon optimisation is a popular approach for improving the chances of producing an MP. Advantages of codon optimisation include increased yield and an ability to produce a protein in cheaper expression systems. There are significant disadvantages to this approach. Synonymous codon changes can lead to protein mis-folding and altered substrate specificity. Consequently, care should always be taken to ensure that the conformation and function of the subsequently produced protein is not altered [40].

Choice of expression vector is another important consideration when designing constructs. A list of the commonly used vectors in the MPL is included in Table 2. The pOPIN vector system has been particularly useful due to the flexible range of tagging options. pOPIN vectors were adapted from pTriEX and modified to enable HTP-screening of protein constructs using a range of purification tags in bacteria, insect and mammalian cells [41]. Utilisation of the proprietary In-Fusion system (TakaRa Bio Europe) enables 192 constructs to be cloned and tested for expression in under 10 days [42]. In-Fusion is a ligation-independent cloning (LIC) method that avoids the need to digest inserts with restriction enzymes. Traditional restriction enzyme-cloning methods are problematic for HTP cloning due to potential internal restriction sites in the inserts. The In-Fusion process is single stepped (the PCR product is cloned directly into the expression vector), and there is no incorporation of amino acids derived from the cloning process due to the precise nature of the In-Fusion enzyme. Other widely used LIC methods, suitable for HTP-cloning, include Gateway and Gibson assembly [43,44].

pOPIN vectors enable the selection of N or C terminal tags consisting of polyhistidine (his-tag), Small Ubiquitin-like Modifier (SUMO), biotin, Strep, green fluorescent protein (GFP), FLAG, maltose binding protein (MBP) and others. Most tags can be removed with 3C protease or in some cases using Tobacco etch virus (TEV) protease. Typically, the inserts are prepared by proofreading PCR to add overlaps matching the ends of the cut recipient plasmid; these are joined and used to transform *Escherichia coli* (*E. coli*) in a 96-well format. Minipreps of the constructs can be made using commercial HTP kits, by robot or manually using a multichannel pipette and vacuum manifold. Constructs can be quickly verified by PCR, to avoid costs associated with sequencing which can be carried out after narrowing down the constructs based on MP expression. Occasionally, constructs are resistant to protease cleavage and in these cases, we have found an extended linker region between the protein of interest and the protease cleavage site prevails.

### 2.2. Recombinant Membrane Protein Expression

Fifteen years ago, the most commonly used expression system was *E. coli* followed by yeast; however, the move towards eukaryotic MP structural biology has seen a shift firstly towards insect cell expression systems and more recently mammalian cell expression systems. The shift towards eukaryotic targets and expression systems correlates well with an increase in the number of eukaryotic structures (Figure 1). Traditionally, milligram quantities of an MP have been required for crystallography. The requirement of high protein yields led to the use of recombinant overexpression, rather than natural sources, as the method of choice when producing MPs. The most popular and well characterised bacterial expression system for MPs is *E. coli*, but *Lactococcus lactis* is also a suitable expression system [48]. Advantages of bacterial expression include existing effective tools for genetic manipulation, the low cost of production, rapid growth rates and high cell densities. However, many MPs have proved to be toxic to the host or are incorrectly folded when overexpressed. The accumulation of mis-folded protein is potentially due to high transcription levels from the T7 promotor leading to the saturation of the Sec translocon. Saturation of the Sec translocon and the overloading of the bacterial quality control system in turn lead to the accumulation of the expressed MP in inclusion bodies. [48,49]. Efforts to mitigate this have led to the development of several *E. coli* strains [49,50,51].

Generally, high copy vectors have been avoided when producing eukaryotic MPs due to leaky expression (even in non-expression strains such as TOP10). Glucose can be included in the media or pLacI strains used to avoid leaky expression. Alternatively, Studier’s non-inducing media are a good option to prevent leaky expression [52]. The possibility of toxicity/leaky expression should be considered when cells grow slowly before induction. Autoinduction is convenient for HTP protein expression but may not be optimal for MPs as autoinduction normally happens in the late log/stationary phase and varies depending on oxygenation level. *E. coli*-based MP expression is also affected by temperature, incubator shaking speed and the type and fill volume of the culture vessel. Ideally, expression should be induced at mid log (OD of 0.4–0.8 in LB, somewhat higher in high-density media such as terrific broth or Power Broth), when the cells are at their most healthy stage and best able to tolerate overexpression of MPs.

Many eukaryotic membrane proteins fail to express well in *E. coli*, so it is often necessary to use a eukaryotic host. Whilst some such targets will need to be expressed in higher eukaryotes (insect or mammalian) to obtain good expression levels, yeast is sufficient for others. The main benefit of yeast over higher eukaryotic cell culture is that it is much cheaper—an important consideration when many litres of cell culture may be required. The two main yeast platforms are *Pichia pastoris* and *Saccharomyces cerevisiae* [53,54]. Of these, *Pichia* can give higher expression levels but is initially more time-consuming due to the need for extensive screening to identify a high-expressing clone. The Saccharomyces system, however, uses a plasmid capable of episomal replication, making it convenient for HTP expression trials.

Insect cells are the second most popular expression system for MPs. Although significantly more expensive and time consuming than *E. coli* or yeast, they are an effective system for the expression of eukaryotic MPs, particularly GPCRs. Sf9 cells derived from *Spodoptera frugiperda* and Hi5 cells derived from *Trichoplusia ni* are the most common cell lines used. Recently, new cell lines such as ExpiSf have been derived which enable MP expression at high cell densities. The gene of interest is usually delivered using the baculovirus expression vector system (BEVS), but it is also possible to transiently transfect insect cells [55,56,57,58]. Numerous modifications have been made since the introduction of BEVS over thirty years ago. Many new baculovirus vectors have been developed such as Bac-to-Bac, FastBac, FlexiBAC, flashBAC and MultiBac [59,60,61]. A disadvantage of the FastBac system is the use of *E. coli*-generated bacmid which causes the incorporation of bacterial elements resulting in unstable constructs. Alternatively, the flashBAC system utilises a homologous recombination event to generate baculovirus within insect cells avoiding the incorporation of bacterial elements. The flashBAC system is rendered non-viable, via a site specific insertion into ORF1629, unless rescued by a recombinant formation step where the gene of interest is also inserted [62]. Insect cell expression systems can perform post-translational modifications such as glycosylation, correct disulphide bond formation and having a lipid composition more akin to mammalian systems. However, insect cells struggle to produce large quantities of well-folded, functional MP [63].

Expression in mammalian cell systems such as human embryonic kidney (HEK) 293 and Chinese hamster ovary (CHO) is rapidly becoming the preferred way of expressing human MPs [64,65,66]. Transient transfection methods are an efficient way to rapidly screen constructs on a small scale [67]. However, for large-scale production the amount of DNA required could be as much as 10 mg, making MP production expensive. In these situations, stable cell lines become a much more effective way to produce MPs. The lentiviral approach is particularly effective, with almost 100% of cells transduced, although this method requires a biological containment level 2 facility due to the infectious nature of the lentivirus when generating a stable cell line [68].

### 2.3. Fusion Tags and Resins for MP Purification

**Figure 2:**
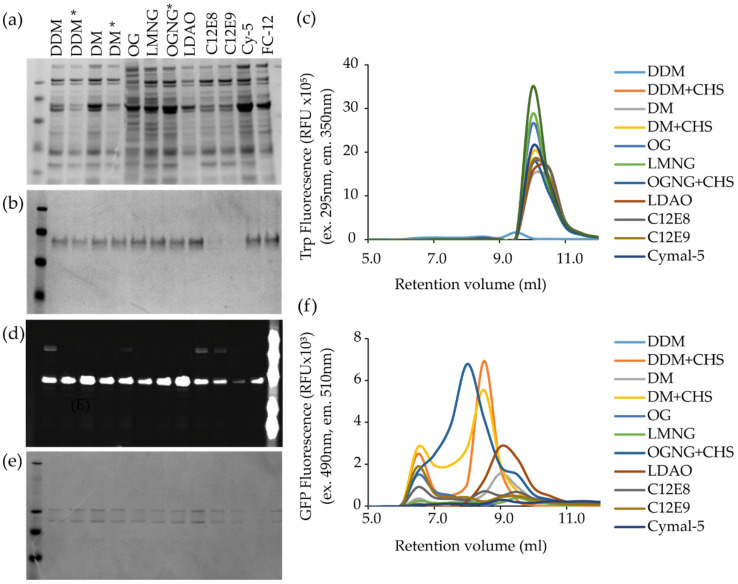
Example sodium dodecyl sulphate polyacrylamide gel electrophoresis (SDS-PAGE) and fluorescence size exclusion chromatography (FSEC) results from a small-scale detergent screen for a bacterial transporter (**a**–**c**) and bacterial membrane kinase (**d**–**f**). A SRT-300 (Sepax Technologies Inc.) high-pressure liquid chromatography column was used for FSEC. This has the advantage of tolerating higher pressures than standard SEC columns, enabling faster flow rates at 4 °C. 96 samples can be processed in 24 h. (**a**) Purification in 12 different extraction conditions of a monomeric bacterial transporter (35 kDa) using Ni-NTA resin. (**b**) Purification of the same bacterial transporter using Talon resin (cobalt). (**c**) FSEC profiles using tryptophan fluorescence (excitation at 290 nm, emmission at 350 nm) for purifications shown in panel b. (**d**) Purification in 12 extraction conditions of a multimeric bacterial histidine kinase. SDS-PAGE gel imaged using GFP fluorescence and (**e**) Coomassie staining. (**f**) FSEC profile using GFP fluorescence (excitation at 490 nm, emission at 510 nm) of purification in panels (**d**) and (**e**). Peaks correspond to monomeric and dimeric protein.

Fusion proteins are commonly used to aid purification of an MP; they can be small such as the his-tag or more complex such as GFP or MBP [63]. Fusion proteins have been used as affinity tags, enhancers of solubility, conformational stabilisers and reporter proteins. One of the most common tags, his-tag, is comprised of between 6 and 10 histidine residues. In order to purify his-tagged proteins, a number of immobilised metal ion affinity (IMAC) resins are available. These resins consist of a solid support coupled to a matrix that immobilises metal ions. Matrices include iminodiacetic acid (IDA), nitrilotriacetic acid (NTA) or carboxylmethylaspartate (CMA). These directly co-ordinate metal ions. The most frequently used metal ions are nickel (Ni^2+^) or cobalt (Co^2+^). TALON cobalt resin is based on a CMA matrix and is particularly effective for MP purification [29,69,70,71]. TALON has a lower affinity for polyhistidine than Ni-NTA; thus, the protein can be eluted at lower imidazole concentrations. Less non-specific binding compared to Ni-NTA is also observed as shown in Figure 2 showing a set of samples purified with (a) Ni-NTA and (b) TALON.

Strep-tags are another commonly used fusion peptide that yields highly pure samples. This tag is designed based on the high affinity binding of biotin to streptavidin resin, such as the StrepII tag consists of eight amino acids (WSHPQFEK) which shows high affinity to Strep-Tactin^®^ resin (K_d_, 1 µM), a derivative resin based on streptavidin [72]. Additional optimisation of the Strep-tag system has led to the use of twin-strep tags. Twin-strep tags offer higher specificity of the protein to the Strep-Tactin resin, yielding purer samples. A benefit of Strep-tags compared to traditional His-tags is the use of biotin or desthiobiotin to elute the desired protein, which is milder than the high concentrations of imidazole traditionally used to elute his-tagged proteins.

Other high affinity tags include Avi-tags which consist of a unique fifteen amino acid sequence (GLNDIFEAQKIEWHE) that can be biotinylated on the lysine using the biotin ligase enzyme BirA from *E. coli* [73]. Biotinylated Avi-tagged protein has a high affinity for streptavidin, the precursor to strep-tactin used for strep-tags. High purity Avi-tagged protein can be obtained in a single step using the same procedure as strep-tagged proteins [74].

Alternative protein capture approaches use nanobodies which are highly stable and potent single domain containing fragments of camelid antibodies [75]. Nanobodies have been produced against GFP [76]. They can be tagged with a highly specific classical tag (e.g., strep-tag) and pre-bound to a specific resin before applying the GFP-tagged protein sample. The column becomes visibly green, confirming binding of the GFP-tagged protein. Elution of the sample can be achieved either by on-column cleavage in the presence of a relevant protease, or by elution of the nanobody with a suitable eluent.

The use of IMAC resins has been associated with the purification of several contaminants which can pose problems for MP structural biologists. Among the most common is AcrB, a bacterial proton motive force-dependant multidrug efflux pump that has been solved by numerous groups when attempting to purify other MPs [77,78,79,80]. AcrB has a histidine rich sequence and readily crystallises in common MP crystallisation screens at concentrations that are hard to detect by sodium dodecyl sulphate polyacrylamide gel electrophoresis (SDS-PAGE). AcrB contamination was first reported in 2008 [77], but is still an issue in the MP community. AcrB contamination was quickly identified in three recent projects brought to the MPL. Inclusion of a tag cleavage step and reverse IMAC is the simplest way to ensure that contaminating proteins such as AcrB are not present. The cleavage and reverse IMAC steps remove the need for extensive washing during the first IMAC step, which can be detrimental to MP stability. Identification of SDS-PAGE MP by tryptic digest and tandem mass-spectrometry (MSMS) is useful especially if an MP is similar in size to the contaminant. Comparing unit cells and space groups with known AcrB structures can avoid spending time optimising AcrB crystals. Expression in a ΔAcrB *E. coli* variant or purification with specific tags such as FLAG or twin-strep can avoid contamination [81]. We have also observed the co-purification of cytochrome c oxidase complex with some user projects when using IMAC. The chances of cytochrome c oxidase complex contamination are especially pronounced when the MP is expressed in *Lemo21(DE3)*. Fortunately, cytochrome C oxidase has a characteristic colour and thus is readily identifiable during purification. OmpF, ferritin and NONO are other common IMAC contaminants found in *E. coli*, insect cells and HEK cells, respectively [79,82].

## 3. Stabilisation of Membrane Proteins for Structural Studies

Stable protein is a prerequisite for any structural technique. Protein instability can arise from either conformational and/or compositional factors. The protein of interest must survive the purification process and be stable for long enough to either crystallise or be applied to a sample mount for EM. Specific factors vary somewhat depending on the structural approach taken. Cryo-EM is more forgiving of conformational instability enabling multiple conformations of a protein to be solved at atomic resolution [83]. Conversely, the very nature of a crystallisation experiment means that individual crystals only relate to one structural conformation. Either way, MPs present an additional compositional challenge, as in order to study them at atomic resolution, they must be removed from their native environment. Extracting an MP from its native environment in order to study it is an empirical process, affected by expression levels and the propensity of an MP to be misfolded. To mitigate these problems, it is sensible to screen multiple MP constructs that consist of natural variants across different species. Alternatively, engineered sequences can be screened, leading to multiple constructs with different truncations, fusion constructs and/or mutations [84,85,86]. Identifying the most suitable constructs can be time-consuming. For example, it can take 6–12 months to thermostabilise a GPCR [86]. It is, therefore, important that screens are in place to identify suitable constructs as quickly and efficiently as possible. In many cases, especially GPCRs, screens often take the form of a binding assay using a known high affinity, radiolabelled ligand. When direct assays are not available, other reporters can be used such as GFP to identify well expressing, correctly localised constructs prior to purification [87]. Genetic fusions of MPs and GFP have been generated for constructs expressed in bacteria, yeast, insect and mammalian cells [45,88,89,90]. The GFP tag can also be used to track protein through the purification process and can be used as a tag for purification or as part of a pre-crystallisation screen [90,91,92].

### 3.1. Use of Encapsulation Agents to Stabilise Membrane Proteins

MP purification is traditionally focused on identification of the most suitable detergent for purification before exchange into a shorter chain detergent prior to crystallisation. Detergents are still among the most common tools used to extract MPs from membranes, and choice of detergent is a critical step when trying to purify an MP for crystallisation or cryo-EM [93,94]. Over the last twenty years, new generations of detergents have been developed. These include fluorinated surfactants where fluorine is incorporated into the alkyl tail leading to a more rigid detergent, with increased hydrophobicity and much lower critical micelle concentration (CMC). However, these detergents tend to poorly solubilise lipids and other hydrophobic cofactors [95]. Alternatively, the alkyl chain can be modified to produce branched detergents that are better lipid mimics such as the neopentyl maltoside and neopentyl glucoside classes of detergents [96,97]. Lauryl maltoside neopentyl glycol (LMNG) has been demonstrated to be an effective detergent for crystallisation of GPCRs in lipidic cubic phase (LCP) [89,98]. LMNG is also an effective detergent for the stabilisation of MPs for cryo-EM [99].

With such a wide range of different detergent options available and the importance of working with stable correctly folded protein, it is necessary to identify the best combination of constructs and detergents with which to work. For constructs with a GFP tag, solubilised cell lysates can be analysed by FSEC [45,87,88,92]. More recently, this approach has been expanded to screen a wider range of detergents [100]. Where HTP-SEC is unavailable, filter plates can be used to crudely separate differently sized MPs, but these are less suitable for large MPs [101]. Alternatively, a simple extraction assay can be applied to assess folded state [102]. It is important to note that the most suitable condition is often not the condition that extracts the largest amount of MP. A prime example is with Fos-Choline-based detergents that readily extract MPs, often in an inactive and mis-folded state [102]. This is particularly important when working with GFP tagged MPs expressed in eukaryotic expression systems such as yeast, insect cells and mammalian cells as GFP can remain fluorescent regardless of whether the MP is correctly folded [102].

In the MPL, we use a combination of a simple extraction assay (including harsh control detergents such as SDS or Fos-Choline), coupled with small-scale purification before analysis by SDS-PAGE and FSEC. The GFP signal from the IMAC elution is used to ensure that only detergents that have effectively purified the MP of interest are screened by FSEC. This way we can screen multiple detergents or MPs in parallel in a 96-well plate format in 24 h (Figure 2a–f). Care should also be taken to ensure that the detergent does not inhibit the protease used for tag removal [103].

Although detergents have been an effective way to solubilise MPs, their use can lead to a loss of protein–protein and protein–lipid interactions, potentially destabilising the MP. Some lipids are critical for MP function, and thus, removal would lead to inactive protein, as is the case for two mammalian glucose transporters [71]. The destabilisation of an MP can sometimes be mitigated by identifying a suitably mild detergent, but mild detergents are not always compatible with the proposed downstream experiment. Small chain detergents are typically required to obtain higher resolution structures via X-ray crystallography, but these can be very destabilising to the MP. Common detergents used for X-ray crystallography such as DDM or LMNG can also be problematic when trying to distinguish a protein particle from detergent by negative stain EM as demonstrated by grids that were prepared of the citrate transporter, CitS, in DDM and DM [104]. Free detergent micelles can be removed prior to the preparation of grids to reduce background interference [99].

To avoid some of the problems associated with detergents, other systems have been developed that maintain MP stability in solution and support an environment more analogous to the membrane. These include membrane scaffold proteins (MSPs), Saposin lipoprotein nanoparticles (Salipro), Styrene maleic acid co-polymer lipid particles (SMALPs), amphipols and peptidiscs [105,106,107,108,109]. All have been shown to be effective tools for cryo-EM sample delivery [110]. Additionally, SMALPs can be used to deliver MP samples into the lipidic cubic phase for crystallisation [111].

MSP nanodiscs are one of the most used non-detergent approaches for stabilising MPs. They are derived from human serum lipoprotein A1 and with additional phospholipids provide a native phospholipid bilayer environment, thereby retaining the functional requirements of the incorporated MP [44]. Various sizes of MSP nanodiscs are available ranging from 8–16 nm in diameter. They are soluble, non-covalent assemblies which can self-assemble MPs for functional and structural studies [112,113]. Larger MSP-based nanodiscs face challenges with sample heterogeneity as multiple copies of an MPs can be enclosed within. To overcome this, covalently circularised nanodiscs (cNDs) of 50 nm size were recently developed [114,115,116]. Other large nanodiscs include DNA corralled nanodiscs, which have a 45–70 nm disc size and are mainly used for functional characterisation of MPs [115,116,117]. Another approach is based on Saposin A (SapA), a lipid binding protein which forms a scaffold that surrounds disc-like nanoparticles comprising lipids and MPs. The advantage of saposin lipo protein or Salipro technology is that the SapA scaffold flexibly accommodates variably sized MPs [106]. A recent methodology called DirectMX was developed to reconstitute fragile MPs directly from human crude cell membranes using the Salipro technology [118].

Amphipols are specifically designed amphipathic polymers that provide another tool for MP encapsulation [108]. Amphipols cover the hydrophobic membrane-spanning regions of MPs with a thin layer of surfactant [119]. Typically, detergents are used to first solubilise an MP before exchange into amphipols. Compared to detergents, amphipols are much less likely to strip away lipids or disrupt protein–protein interactions, and lipids can rebind after amphipol exchange [120]. However, due to the way amphipols interact with the surfaces of an MP, they can disrupt conformational changes that may occur in the transmembrane domain. This reduction in conformational movement is known as the “Gulliver effect” and may explain the association between SERCA1 inaction and increasing stabilisation by different amphipols [121,122]. A wide variety of amphipol classes exist that include both ionic and non-ionic derivatives, but the most widely used is A8-35. Other amphipol derivatives include glucose-based non-ionic amphipols (NAPol), Nvoy, sulphonated amphipols (SApols) and Tris-based non-ionic amphipols (APols) [122,123,124,125]. Both A8-35 and some PMAL amphipols have shown reduced MP activity at acidic pH, or in the presence of multivalent cations such as calcium [122]. A8-35 and PMAL amphipols are both incompatible with MP cell-free expression but have been used to encapsulate MPs including ion channels, transporters and enzymes that have been solved by cryo-EM [89,126,127,128]. The non-ionic NAPol is soluble across a wide pH range and along with Nvoy is compatible with cell-free synthesis of MPs [129]. Amphipols have also been used to stabilise an MP prior to crystallisation in LCP [130].

SMALPs, also known as lipodiscs, are another example of a polymer approach toward the stabilisation of extracted MPs. A wide range of co-polymers exist that include: Styrene maleic acid (SMA), polystyrene-*co*-maleimide (SMI), poly-diisobutylene-*co*-maleic acid (DIBMA) and other derivatives of SMA for solubilisation in the purification process. SMALPs enable direct solubilisation of an MP without the requirement for detergent. SMA creates stable nanoparticles 10-11nm in diameter, containing an MP in a lipid bilayer that is suitable for downstream structural and biochemical studies [109,131,132,133]. SMALPs have been used to successfully encapsulate transporters, ion channels, enzymes, receptors and large complexes [131,134,135,136,137,138,139,140,141]. A drawback of SMA is that it is pH sensitive and has a high negative charge. Maleic acid will be protonated in acidic conditions, making the polymer insoluble, and limiting the use of SMA to proteins stable at basic pH [142]. Styrene-*co*-maleimide (SMI), which is positively charged, and the zwitterionic SMA (zSMA) have been developed for a better performance in acidic conditions and in the presence of divalent cations [142,143]. DIBMA (Di-isobutylene maleic acid) is another alternative [144]. Unlike aromatic SMA, aliphatic DIBMA has only a mild effect on the lipid acyl chain order; thus, DIBMALPs are compatible with optical spectroscopy in the wide UV range. DIBMALPs can tolerate low millimolar concentrations of divalent cations accelerating solubilisation by several fold, which helps in functional biophysical assays [145,146,147]. SMA-PAGE combines the MP encapsulation of SMALPs with native gel electrophoresis to separate MP complexes along with surrounding phospholipids in their native state [137,148]. MP-SMALPs can be extracted from gel bands and analysed using EM and mass spectrometry.

Peptidiscs are another non-detergent alternative. The peptidisc approach of reconstituting MPs is rapid and cost effective as it does not require scaffold proteins of various lengths or precise amounts of lipids. It only requires a short amphipathic bi-helical peptide (NSPr) separated by a proline residue, of which multiple copies wrap around the target protein. The reconstitution can be achieved by “on-column”, “in-gel” or “on-bead” approaches and MPs later purified by tag-based affinity chromatography [105]. An alternative “on-gradient” approach can be used to reconstitute a detergent solubilised MP [149]. MPs reconstituted into peptidiscs have been solved by cryo-EM and include the MscS ion channel and MsbA transporter [149].

### 3.2. Genetic Engineering of MPs to Improve Protein Stabilisation

GPCRs were the initial focus of genetic engineering approaches focused on improved MP stability through the introduction of fusion proteins such as T4 lysozyme or BRIL. The first of these led to the first human structure of a GPCR; the β_2_ adrenergic receptor and the method has been developed into a standardised approach that can be applied to other GPCRs and MPs [85,150]. Alternative approaches focus on thermostabilisation through the systematic replacement of native amino acid residues with alternatives such as alanine or leucine. This approach led to the structure of the β1-adrenergic receptor [86,151]. A recent alternative method is a sequence consensus approach that bases stabilisation on conserved residues between homologues [152]. Directed evolution has also been used to produce GPCRs with enhanced expression and stability in detergents [153].

### 3.3. Chaperones and Ligands to Aid MP Stabilisation

MPs may also be stabilised by the inclusion of ligands or chaperones that have a strong affinity for the MP of interest. This approach is often used in tandem with other stabilisation methods. The simplest approach is to include natural substrates, antagonists or agonists. This may lead to preferred conformations but can limit the ability to solve the structure of active MPs as has been the case for most GPCR structures to date. Recently, the development of mini-G-proteins has enabled the capture of active states using crystallography and cryo-EM [154]. If specific functional ligands are unavailable, then there are a number of approaches that can be taken. An early successful approach was to use antibody fragments as co-crystallisation chaperones [155,156,157]. Designed ankyrin repeat proteins (DARPins) have also been used [158,159]. More recently, nanobodies have been highly successful. Since the first nanobody-MP structures have been solved, newer methods to develop nanobodies have been introduced such as in vitro display, which does not require the use of costly animal hosts that are subject to regulatory and ethical approval [160,161]. Directed evolution can also be used to enhance the potency of identified nanobodies. Nanobodies have also been proposed as novel therapeutics [162]. In addition to modulating protein functions, these antibodies can provide useful crystal contacts that can aid crystallisation or can be used to generate megabodies that can enhance the resolution of structures solved by cryo-EM, especially for small MPs [163,164].

### 3.4. Assessing the Quality of Purified MPs

Once an MP is expressed and purified, an assessment should be made as to the suitability of the MP for structural biology-based applications. Suitability will depend on an MP’s functional, conformational and compositional stability. Ideal characterisation techniques will use a minimal sample and provide information that addresses the stability of the MP at concentrations appropriate for the chosen technique.

**Figure 3:**
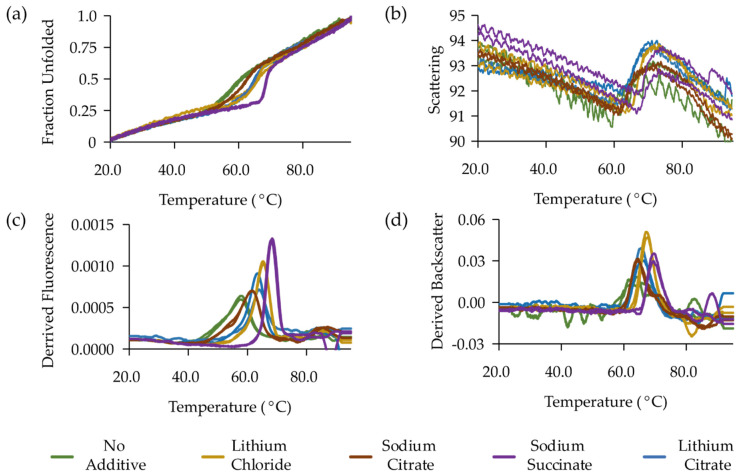
Thermal unfolding of vcINDY in the presence of different substrates measured by intrinsic tryptophan fluorescence and backscatter. (**a**) Thermal denaturation curves depicting fraction of protein unfolded. (**b**) First derivative of (**a**) reporting maximum T_m_ for each additive. (**c**) Scattering curves for each additive. (**d**) Derived scattering curves with the maximum reporting T_agg_.

Assessment of MP function is not always straightforward and may require months of optimisation to generate interpretable results. However, production of functional protein is an essential prerequisite for any structural study. At the early stages of the MP production process, suitable pseudo functional alternatives can be explored that can act as a substitute for a full functional assay. One such possibility is to use differential scanning fluorimetry (DSF). This method is based on the measurement of protein unfolding using a fluorescent dye. Unfolding can be induced by heat or with denaturing chemicals. A comparison can be made between the midpoint of the unfolding event in the presence and absence of ligand [165,166]. The original DSF method used a dye that bound to hydrophobic regions of a protein that are exposed during unfolding. However, this approach is not suitable for MPs, and alternative methodologies were developed that rely on cysteine modification [167,168,169]. These methodologies are cheap and use minimal sample volumes but are unsuitable for proteins purified in reducing conditions. An alternative is to use a direct approach such as intrinsic tryptophan fluorescence to measure MP unfolding and backscatter to assess precipitation. NanoDSF instruments such as the Prometheus (Nanotemper) provide an ideal platform to measure this. Here, we present an example where a range of substrates stabilise a dependant dicarboxylate transporter vcINDY (Figure 3). The preferred substrate, succinate, stabilises vcINDY by 10 °C compared to 4 °C from the less readily transported citrate. Preference for succinate is also reflected in the onset of precipitation.

The compositional stability of an MP in detergent is often assessed early in the purification process via FSEC. More recent approaches such as nanoDSF and in situ dynamic light scattering can also be used to access the effect of encapsulation reagents on the stability of an MP [102,170,171]. Negative stain is another method that can be used to assess the compositional stability of MPs but is limited by sample throughput but does have the advantage of using nanograms of sample. First developed in the late 1950s, negative stain EM (electron microscopy) was used to obtain the first high resolution structures of T2 bacteriophages [172]. Preparing negative stain grids is quick and a simple process; thus, assessment of protein homogeneity/heterogeneity can be made quickly using a minimal sample [173]. Negative stain samples are prepared by coating a carbon support with the protein of interest before applying a charged, electron dense, heavy metal salt (the stain). High-resolution (~20 Å) images are obtained by the difference in electron density between the highly dense stain and much lower electron density of the protein sample. The most commonly used stains for MPs tend to be uranyl acetate and uranyl formate, but other alternatives exist [174]. Like all structural techniques, significant optimisation is often necessary if higher resolution data are required. However, using negative stain EM can be a powerful approach that can be used to assess protein quality using a minimal MP sample.

Conformational stability encompasses changes to MP secondary, tertiary and quaternary structure. Secondary and tertiary structural changes can be assessed by circular dichroism either using lab-based instruments or synchrotron radiation [175,176]. Ion mobility mass spectrometry can also be used to assess tertiary structural changes [177]. To access quaternary structural changes, size exclusion chromatography coupled to multiangle light scattering (SEC-MALS) can be used. The SEC-MALS system is equipped with three detectors. Ultra-violet (UV-280 nm), multiangle light scattering (MALS) and refractive index (RI). The data from these detectors can be used to calculate the oligomeric state of an MP, the amount of detergent associated, and the amount of free detergent by using co-polymer analyses. The MALS instrument is equipped with a right angle light scattering (RALS) detector and a low angle light scattering (LALS) detector, set at 90° and 7° to the incident beam, respectively. The light scattered by a protein sample is used by the system to determine the molecular weight of the protein. The LALS detector can accurately measure the molecular weight of samples of all sizes. However, the intensity of scattered light will be lower for smaller samples at the angle of the LALS detector. Small samples uniformly scatter light; thus, to maximise the signal to noise ratio for smaller samples (less than ~15 nm), the RALS detector can be used. The RI detector is used to measure the concentration of each component in the sample. This includes protein and detergent as well as any post-translational modifications such as glycosylation. By using a co-polymer analysis, the amount of protein, glycosylation, protein-associated detergent as well as the free detergent micelles can be calculated [171,178,179].

**Figure 4:**
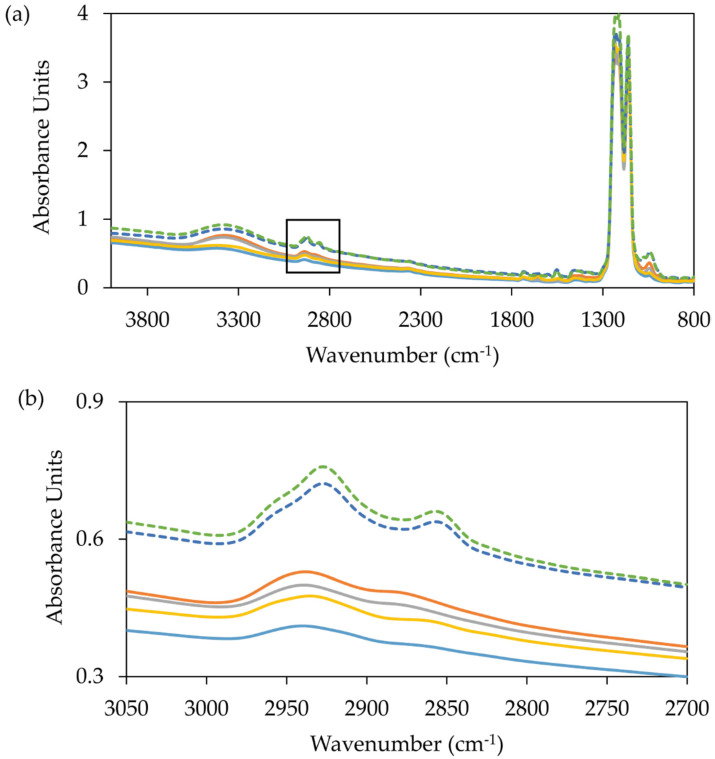
MIR spectrum of six different membrane protein purifications in octyl glucose neopentyl glycol (OGNG) with cholesteryl hemisuccinate (CHS) from ZMPSTE24 (solid lines) and a membrane receptor (dashed lines). (**a**) Full MIR spectra and (**b**) magnified MIR spectra (3050–2700 cm^−1^). Using a standard curve for a given detergent the amount of detergent in a sample can be quantified. In four of these cases (blue, yellow, grey and orange) detergent content is low (between 0.25% and 0.5%) and at the detection limit for the method. Low detergent readings are expected for these optimised purifications where detergent content is minimised throughout the purification. In the case of the membrane receptor, detergent concentrations are much higher ~2% (dark blue and green) as indicated by the peak at 2850 cm^−1^.

Quantification of the free detergent content of an MP sample is an important parameter to measure as the free detergent content can affect the crystallisation of an MP. SEC-MALS is sample intensive and thus not appropriate for low yielding MPs. An alternative method that can be used to quantify total detergent content is based on the quantification of sugars but is not suitable for non-sugar-based detergents such as the polyoxyethelene series [180]. Mid infra-red (MIR) spectroscopy can be used to quantify lipid and detergent content [181,182]. The aliphatic group stretch between 2800 and 3000 cm^−1^ can be used to quantify lipid content, while vibrational frequencies between 2840 and 2870 cm^−1^ can distinguish between lipid and detergent. The Direct Detect (Merck Millipore) enables the measurement of dried down samples using minimal sample volume (2 µL) in a way that is easily accessible to researchers who are not experts in MIR. Standard curves can be prepared in advance for relevant detergents so that sample measurements can be made quickly in a similar way to measuring the concentration of a protein using a Nanodrop spectrophotometer. Full infra-red spectra can be obtained that provide a fingerprint-like snapshot of each MP preparation. The methodology can also be used to assess protein concentration using the amide I region (1600 to 1690 cm^−1^) [181]. A comparison of different MPs and purifications is shown in Figure 4.

## 4. Advances in Sample Preparation and Data Collection

When the MPL was established, crystallography was the only mainstream structural technique available that could be used to solve atomic resolution structures of the majority MPs. At the time, a huge focus had been placed on the miniaturisation and automation of crystallisation screening. New dedicated beamlines at synchrotrons were being built that would better suit the smaller crystals typically obtained for MPs but availability was limited and thus they were highly oversubscribed. Advance ten years and single particle cryo-EM now rivals crystallography as the method of choice for solving MP structures. Availability of suitable electron microscopes is now a major problem. Miniaturising and automatising sample preparation for crystallography are now well established but cryo-EM still lacks consolidated efforts.

### 4.1. Membrane Protein Crystallisation and Data Collection

MP crystallisation is in general no different to the crystallisation of soluble proteins. The overall aim is to identify conditions that will lead to nucleation and subsequent crystal growth. Crystal growth is affected by multiple factors such as temperature, pH, ionic strength, precipitant and protein concentration. As many variables can affect crystal growth, a wide range of conditions must be screened. MP-focused crystallisation screens have been designed based on existing MP crystallisation conditions [183,184]. Most crystallised MPs are detergent solubilised and are either directly set-up using simple vapour diffusion methods (hanging or sitting drop) or exchanged into lipidic environments. Detergent-based crystallisation of MPs leads to type I MP crystals, while lipid-based crystallisation tends to produce type II crystals. Type II crystals also tend to diffract to higher resolution due to the formation of additional crystal contacts in the membrane spanning regions. Consequently, type II crystals have a lower solvent content and are, therefore, more robust [171]. Selection of the right detergents for direct crystallisation is necessary to obtain well diffracting crystals [94,102,185]. Detergents with shorter chains form smaller micelles that result in tighter crystal lattice packing and improved diffraction compared to detergents with longer acyl chains, although these tend to be more efficient for solubilisation and stabilisation [102,186,187]. Control of detergent concentration is also vital as too much detergent can prevent crystallisation and promotes detergent phase separation and detergent crystals. In some cases, a suitable mixture of detergents must be used to alter the protein–detergent complex instead of a single detergent which is sufficient in most cases [188].

The HiLiDe approach to crystallisation sits between the simple direct crystallisation of detergent solubilised samples and lipid-based crystallisation methods. It has been particularly successful for P-type ATPases [189,190]. High lipid and detergent concentrations are systematically screened using detergent solubilised samples prior to setting up crystallisation plates via vapour diffusion. This systematic screening has the advantage of stabilising MPs prior to crystallisation (precipitated protein is removed as part of the screen) and leads to type II crystals which can be easily harvested. HiLiDe tends to require large quantities of samples especially if all suggested conditions are screened. However, we find that when MP yield is limited, screening a smaller number of conditions covering the same range of lipid/detergent concentrations can be an effective way of obtaining initial MP crystals that can be subsequently optimised.

Around a third of crystal structures have been solved using lipid-based crystallisation methods that include LCP, lipidic sponge phase (LSP) and bicelle crystallisation [191,192,193]. LCP and LSP crystallisation are based on the mixing of a solubilised MP (in aqueous solvent) with a lipid usually a monoacyl glycerol (MAG). Different lipidic phases can be achieved depending on the lipid, the ratio of solvent to lipid or by adjusting the temperature. The MAGs used for the crystallisation of MPs in LCP are fatty acids with a glycerol head group linked by an ester linkage to a fatty acyl tail. The fatty acyl tail contains a monounsaturated *cis* double bond. MAGs can be described by the head, neck and tail notation [194]. This is a simplified nomenclature that splits the MAG up into the glycerol head group, followed by the number of carbons before (neck) and after (tail) the *cis* double bond. Adding the number of carbon atoms from the neck and tail region together gives the total length of the fatty acyl chain. The most commonly used lipid is monoolein which can also be described as MAG 9.9. This is because there are nine carbons in the acyl chain before the *cis* double bond and nine carbons after. MAG 9.9 has a fatty acyl chain that is 18 carbons in length. Other MAGs with shorter chains such as MAG 7.7 have also proven to be successful agents for the crystallisation of MPs in LCP. LCP has been a particularly effective method for crystallising small compact MPs, especially GPCRs, but there are also many examples from other MP families [195]. The crystals formed in LCP are mostly micro crystals and difficult to see in the LCP environment. The use of cross-polarised light greatly enhances visibility. Crystals are typically grown in glass sandwich plates but can also be grown in sitting and hanging drop plates. As an optimisation tool for protein samples in LCP, LCP-fluorescent recovery after photo bleaching (LCP-FRAP) can be used as a pre-crystallisation step. LCP-FRAP operates in an automated HTP mode to determine the diffusion rate of an MP in LCP. MPs with a low diffusion rate in LCP-FRAP are less likely to crystallise [14,196,197,198].

The viscous LCP also acts as a highly efficient delivery system for serial femtosecond crystallography (SFX) at advanced X-ray sources, such as free-electron lasers (XFEL), and microfocus beamlines in synchrotrons [199]. XFEL and SFX approaches enable structural information to be obtained from multiple crystals at room temperature with minimal radiation damage. The serial approach eliminates the need to freeze and mount MP crystals [195].

The miniaturisation of crystallisation experiments through the development of nanolitre dispensing liquid handlers, including LCP dispensing instruments, significantly enhanced MP structural biology. Concurrently, the standardization and commercialisation of crystal mounts simplified the screening of these crystals on home and synchrotron X-ray sources [200]. Loop matching crystals is an effective way to improve signal-to-noise ratio as well as aiding the automated systems used to identify crystals at synchrotrons.

The act of preparing a sample for X-ray diffraction-based studies has not changed much over the past fifteen years. Crystals are first cryoprotected, mounted in a crystallisation loop and finally flash frozen in liquid nitrogen. MPs tend to have an advantage over their soluble counterparts and will often crystallise in low molecular weight PEG conditions and thus not need additional cryoprotectants. The presence of suitable cryoprotectants is especially true for MP crystals grown by LCP where PEG 400 is the basis for most crystallisation screens. The mounting of an MP crystal in a loop is still a mainly manual exercise, although there have been developments towards automated mounting. These include the fully automated Crystaldirect approach which relies on using cyclic olefin copolymer (COC) film plates and excising whole wells and the semi-automated shifter system that enables individual crystals to be mounted at 6 x the rate of a standard mounting session [201,202]. COC film plates have also been developed for LCP crystallisation of MPs [203,204,205].

To overcome some of the challenges of mounting protein crystals there has been a push towards a return to in situ data collection. The development of crystallisation plates that have low X-ray backgrounds as well as mounts for such plates on synchrotron beamlines has enabled the rapid identification of initial protein crystals [11,206,207]. Recently, this has been further enhanced with the development of beamlines such as VMXi at Diamond Light Source that is dedicated to screening and collecting data from multiple crystals in situ [208].

The last fifteen years have been witness to rapid developments in synchrotron technology. Many aspects of a macro-crystallography (MX) beamline including optics, sample handling, detectors and software have been optimised to enable faster, automated, data collection. Advances in auto-processing have enabled near realtime feedback [7]. The number of crystals that can be screened per hour has improved from four in 2007 to more than twenty in 2017 and will improve further with faster detectors, improved X-ray centring strategies and automated data collection pipelines. In addition, new types of beamline have been developed such as the now established microfocus technology that enables beam sizes as small as 5 × 1 µm. New developments will allow sub 1 µm beams [6,209,210,211]. Helical data collection strategies have also been successfully employed and enable efficient data collection from rod shaped crystals which are not uncommon MP crystal forms [212,213]. A major advantage of collecting data in this way is the reduction in radiation damage as data collection is not focused in one area of the crystal. Long-wavelength beamlines will help overcome problematic datasets where molecular replacement and standard experimental phasing methods fail [214]. Long-wavelength beamlines can also reveal the presence of metal ions that cannot be detected on standard beamlines [215].

### 4.2. Preparation of Membrane Proteins for Cryo-EM and Data Collection

Developments in cryo-EM over the last decade have had a profound influence on not just the study of MPs but in many other areas of life science. Here, we will briefly summarise the major advances and milestones, but the interested reader is directed to other, more detailed reviews [216,217]. Since the structure of the first MP to be solved by cryo-EM, the ion channel TRPV1, in 2013 [218], there have been 280 structures of MPs deposited in the PDB. The number of structures deposited each year has rapidly increased (Figure 1). The highest resolution structure of an MP now stands at 1.7 Å [219].

This paradigm shift in the study of MPs by cryo-EM has been made possible by the “resolution revolution” [220], which has principally been brought about by the development of direct electron detectors, which provide vastly improved speed and sensitivity compared to previous technology. This advancement, coupled with improvements in data processing (such as correcting for beam-induced movement), automated data collection and image processing and other technical advances in transmission electron microscopy (such as phase plates which increase the contrast of particles in cryo-EM), has provided rapid and unparalleled insight into biological systems [221,222,223].

Despite these advances in data collection and analysis, the major bottleneck for the study of MPs by EM is in sample and grid preparation, the technology for which has not developed at the same pace as for the microscopes. Traditionally, cryo-EM grid preparation involves plunge freezing protein, which has been applied to a grid and the excess blotted away, into liquid ethane. However, this technique has issues with regard to reproducibility of ice thickness [110]. To overcome this, alternative blotting-free methods have been developed, including, but not limited to, instruments such as the SPT Chameleon, which deposits nanolitres of sample onto self-wicking grids which are then plunge frozen [224].

As developments in grid preparation and grid technology (such as gold foil on gold supports to minimise thermal contraction during vitrification [225]) catch up with developments already made to the design and use of electron microscopes, it is likely we will continue to see more structures of MPs solved by cryo-EM. These developments will also allow the study of more challenging targets, such as smaller MPs.

## 5. Conclusions and Future Perspectives

The MPL has been supporting MP structural research for nearly fifteen years. In that time, new atomic resolution structures of MPs have helped to advance the mechanistic understanding of this important class of protein. The deposition rate has increased from around 30–40 unique structures per year to 70–80. This has been achieved either through small, interactive advances such as new reagents for MP encapsulation and improved expression systems, or via large wholesale advances such as the rise of cryo-EM. We are currently seeing a shift towards cryo-EM which will continue over the next five years as improvements to electron microscopes; faster, more efficient direct electron detectors; and associated user-friendly software exert their influence [226]. Crystallography will continue to contribute through innovative beamlines such as long-wavelength data collection, while serial crystallography and X-FEL sources will be a focal point for studying reaction intermediates through time-resolved experiments [227,228]. Some of the similarities and differences between X-ray crystallography and cryo-EM have been highlighted in Table 3.

Every new development in MP structural biology shifts the bottlenecks in a new direction and with accessibility for crystallography and cryo-EM improving that bottleneck is now firmly associated with protein production. Current MP production is costly and time consuming as it is not possible to predict the best conditions that enable conformational and compositional MP stability. Multi-dimensional small-scale screens will overcome this limitation significantly reducing production costs while improving sample throughput.

Biological systems are immensely complex with proteins participating dynamically within large protein complexes and cellular pathways. To help understand the structural basis for these systems, structural biologists have focused on individual components and used environmental mimics such as detergents to isolate MPs of interest. In doing so, we potentially affect the structural and functional integrity of the MP. Detergents can be avoided altogether by using nanoencapsulation agents such as SMALPs or MSPs, thus retaining the lipid environment and reducing the effects of lipid stripping. Single particle cryo-EM can subsequently be used to solve the structure of MPs in these membrane mimics [135,245]. Additionally, small angle X-ray and neutron scattering may provide complementary approaches to help understand the relationship between MPs in those models, while mass spectrometry could be used to look at MPs directly ejected from native membranes [246,247,248,249,250]. Cryo-electron tomography (cryo-ET) is perhaps one technology that will enable conformational changes on the atomic scale to be linked with dynamic and temporal changes observed across whole cells [251]. Cryo-ET is an already established technique commonly used to image viruses and cellular structures, and recent advances have led to near atomic resolution structures of large bacterial and eukaryotic complexes [252,253,254]. Correlative approaches that combine super resolution light microscopy with cryo-Correlative Light and Electron Microscopy (cryo-CLEM), cryo-ET or cryo-X-ray tomography will equally enhance our understanding. Thus, through integrative structural biology-based approaches, data can be combined to provide powerful biological models of cells resolved between 0.1 nm and 100 µm. [255].

## Figures and Tables

**Table 1:** Membrane protein (MP) structures that have been supported by the work of MPL researchers.

MP Target	Target Origin	PDB Accession Code	Year	Publication
PsrABC	Bacterial	2VPW, 2VPX, 2VPY, 2VPZ	2008	[17]
AcrB	Bacterial	2W1B	2008	[18]
MHP1	Bacterial	2JLN, 2JLO, 2 × 79	2008/2010	[19,20]
POT	Bacterial	2XUT	2010	[21]
H1R	Human	3RZE	2011	[22]
ASBT	Bacterial	3ZUY, 3ZUX	2011	[23]
MGL	Bacterial	3RM3, 3RLI	2012	[24]
NavAb	Bacterial	4F4L	2012	[25]
RCE1	Bacterial	3VG9	2013	[26]
IPCT-DIPPS	Bacterial	4MND	2014	[27]
EptA	Bacterial	5FGN	2015	[28]
MraY	Bacterial	5JNQ	2016	[29]
SiaT	Bacterial	5NV9, 5NVA	2017	[30]
PfeA	Bacterial	6R1F, 6I2J	2018	[31]
AR3	Archaea	6S63, 6S6C, 6GUY, 6GUX, 6GUZ	2019	-

**Table 2:** Expression vectors commonly used at MPL. Expression hosts abbreviated as follows: *E. coli* (E), *S. cerevisiae* (S), insect (I), mammalian (M). Many vectors contain a green fluorescent protein (GFP) tag that is used to assess protein expression, localisation and monodispersity before large-scale purification.

Vector	Selection Marker (*E. coli*/Host)	Tagged Terminus	Protease	Tags	Expression Host(s)	Reference
pWaldo GFP 8His ^1^	Kan/Kan	C	TEV	GFP-8His	E	[45]
pDD-GFP2 ^1^	Amp/Uracil dropout	C	TEV	GFP-8His	S	[46]
pOPINEneo 3C-GFP ^2^	Amp/Neo	C	3C	GFP-8His	E, I, M	Addgene plasmid # 53534
pOPINEneo TEV-GFP	Amp/Neo	C	3C	GFP-8His	E, I, M	-
pOPINE-BAP ^2^	Amp/-	C	-	Biotin	E, I, M	Unpublished
pOPINS3C ^2^	Amp/-	N	3C	SUMO	E, I, M	[47], Addgene plasmid # 41115
pOPINF ^2^	Amp/-	N	-	6His	E, I, M	[41], Addgene plasmid # 26042
Popinj ^2^	Amp/-	N	3C	6His-GST	E, I, M	[41], Addgene plasmid # 26045
pOPIN- HALO7 ^2^	Amp/-	N	3C	6His-HALO7	E, I, M	As for pOPINS3C, Addgene plasmid # 41117
pOPINE-3C-HALO7 ^2^	Amp/-	C	3C	HALO7-6His	E, I, M	Addgene plasmid # 41126, unpublished
pOPINEneo-3C-2Strep ^2^	Amp/Neo	C	3C	2StrepII-8His	E, I, M	Unpublished
pOPINEneo-3C-3FLAG ^2^	Amp/Neo	C	3C	3FLAG-8His	E, I, M	Unpublished

^1^ These plasmids were a gift from David Drew; ^2^ these plasmids were a gift from Ray Owens.

**Table 3:** A comparison of the different variables that affect structural studies undertaken by X-ray crystallography and single particle Cryo-EM.

	X-ray Crystallography	Cryo-EM
Protein size range	Average size of solved structures is ~100 kDa [7].	Typically above 100 kDa. Volta Phase plates have been used to boost signal-to-noise for smaller proteins or binders such as Fabs and megabodies can be used to increase particle size [163,217].
Sample heterogeneity	Usually a homogeneous sample is required [229].	Can tolerate some sample heterogeneity but homogeneous samples lead more quickly to higher resolution structures [216].
Sample concentration	Large quantities of pure protein [230]. Typically 100 to 200 µL at 5 to 40 mg mL^−1^.	Small quantities of pure protein [230]. Less than 10 to 100 µL at 0.5 to 5 mg mL^−1^.
Sample preparation	Relies on obtaining diffracting MP crystals which are difficult to obtain. MP must be removed from its native environment. Crystals grown in crystallisation trays, mounted in a loop and cryo-cooled in liquid nitrogen [171].	MP blotted on to EM grids and vitrified in liquid ethane [110,231]. Single particle analysis can be carried out on proteoliposomes providing a more native environment [232].
Screening throughput	High. Typically in 96 well plates, allowing 100s of conditions to be sampled simultaneously [171].	Low. Each condition to be screened must be imaged individually. Negative stain can be used to narrow screening conditions [216].
Collection method	X-ray diffraction of protein in crystalline lattice, typically using a synchrotron source [171]. Microcrystal electron diffraction is an area of increasing interest [233].	Electron imaging in conjunction with a direct electron detector. Energy filters and phase plates may be helpful [234].
Collection throughput	High. Typically, 15–30 crystals per hour [7].	Low. Time taken several orders of magnitude behind X-ray crystallography [235].
Data Analysis	Quick and highly automated. Complete datasets can be collected in seconds. Many synchrotrons have automated processing pipelines integrated into the data collection process [7]. Well established software suites such as CCP4i2 to aid the crystallographer [236].	Slow. Reconstructions from 1000s of single images can take many days. Processing pipeline can be automated. Software packages to analyse data less established but constantly improving. Examples include RELION and cryoSPARC [237,238].
Structure-based drug design	Routine, high resolution and high throughput. Well established for GPCRs [13,239].	Currently lacks reproducibility, quality and throughput. Ideally requires protein structures at a resolution of less than 3 Å [240,241].
MP conformational flexibility	Each crystal form relates to a single rigid MP conformation.	MP can be in different conformations, which can be identified during processing (but also impede processing) [216].
Ion identification	Generally straightforward depending on resolution. Long-wavelength beamlines enables sodium ion to be distinguished from a potassium ions [228].	Difficult to identify some anions ions in maps due to negative scattering factors [242]. Electrostatic potential maps may help to overcome this [243].
Resolution	Typical range between 1.5 Å and 3.5 Å. For MPs crystallised in LCP Sub 2.5 Å are common. Highest resolution structure currently a yeast aquaporin at 0.88 Å, PDB: 3ZOJ [244].	Typically, 2.5–4 Å are common including some smaller membrane proteins [217]. Highest resolution structure currently the β3 GABA_A_ receptor homopentamer at 1.7 Å, PDB: 7A5V [219]. EM density maps can identify protein and ion charge states [242].
